# Supplementary material for: Conserved roles of C. elegans and human MANFs in sulfatide binding and cytoprotection
Source: Nat Commun. 2018 Mar 1;9:897. doi: 10.1038/s41467-018-03355-0 (PMC5832864; doi:10.1038/s41467-018-03355-0)
Supplement: Supplementary file 1 — Supplementary Information [file 41467_2018_3355_MOESM1_ESM.docx]

**Supplementary figures and legends**

**Supplementary Figure 1 A forward genetic screen identified *C. elegans manf-1* in regulating *hsp-4*::GFP.** **a** Workflow representation of chemical EMS-induced random mutagenesis of the *C. elegans* strain expressing *GFP* driven by the ER stress-inducible *hsp-4* promoter. Isolated F2 mutants carry homozygous mutations causing constitutive *hsp-4*::GFP expression. **b** Map of *C. elegans* chromosomes showing DraI SNP polymorphisms that differ in the Hawaiian strain CB4856 and the Bristol N2 strain. Two recombinants resulting from crosses between CB4856 and *dma1* in the N2 background mapped the fully penetrant *dma1* mutation on chromosome IV between two SNPs Y38C1BA (-16 cM) and F42A6 (-5 cM). **c** Bulk segregant analysis showing strong linkage of *dma1* (the mutant #5) to the SNP C45G7 (-7.99 cM) on chromosome IV without apparent linkage to chromosome V. 6 independent *hsp-4*::GFP mutants were analyzed for comparison. **d** Sanger sequencing confirming the C>T transition mutation in *Y54G2A.23* (named *manf-1*), identified from the whole-genome sequencing of *dma1* mutants near the C45G7 locus. **e** Quantification of effects of ER stress-inducing tunicamycin on wild type and *manf-1(tm3603)* mutant animals. Shown are numbers of progeny from animals in 72 hrs post L4 after 1 or 24 hrs treatment of tunicamycin on NGM plates. N=5 biological replicates, *** P<0.001, ANOVA. Error bars: S.D. **f** Representative GFP fluorescence images of *C. elegans* adults (with the *manf-1* null mutation tm3603 or RNAi) bearing constitutively high levels of *hsp-4p::GFP* expression. Scale bar, 50 µm.

**Supplementary Figure** **2** ***C. elegans* and Human** **MANFs but not its paralog CDNF bind to sulfatide.** **a** Primary sequence of the *C. elegans* MANF-1 showing all structural motifs found in all metazoan MANF orthologs, including the N-terminal signal peptide and the ER retention signal motif at the C-terminus, and the redox CXXC motif at the C-terminal part of the protein. **b** Homology-based structural modelling (Swiss MODEL) of the *C. elegans* MANF-1 against the solved crystal structure of the Hs-MANF showing that the *C. elegans* ortholog contains saposin-like fold at the N-terminus and SAP-like fold at the C-terminus. Causal S75L mutation together with K104L (K112L in Hs-MANF) are located in the N-terminal saposin-like domain. **c** Cladogram indicating the evolutionary relationship and conservation of the MANF protein family in animals. **d** Schematic illustrating the various MANF domains, tags and critical mutations in cDNA constructs used in this work. Purification of recombinant Hs-MANF and K112L mutants from *E. Coli*. **e** Lipid overlay assay of purified recombinant *C. elegans* MANF-1, Hs-MANF and Hs-CDNF using Echelon sphingostrip membranes. Only Ce-MANF and Hs-MANF can bind to sulfatide. **f** Amino acid sequence alignment of MANF orthologues from several major animal species showing high levels of conservation of MANF at the protein sequence level.

**Supplementary Figure** **3 Importance of sulfatide for binding to the** **N-terminus of MANF and uptake by *C. elegans*.** **a** Limited proteolytic analysis by trypsin-mediated digestion showing protection of Hs-MANF by sulfatide (with indicated molar ratio of MANF:sulfatide at 1:5 and 1:10) compared with that of Hs-MANF(K112L) mutants. The protein cN-II (Cytosolic 5'-Nucleotidase II) that does not bind to sulfatide as control was cleaved by trypsin unaffected by presence of sulfatide (with indicated molar ratio of cN-II:sulfatide molar ratio at 1:10). **b** Ion extracted chromatograms showing peak intensity for N-terminal peptide after limited proteolysis of Hs-MANF. The peptide was detected only in the presence sulfatide, indicating protection of this region after ligand binding. Data were obtained from chymotrypsin-based peptide mass fingerprinting. **c** Fluorimetry analysis of purified Hs-MANF with various doses of sulfatide. Thermal denaturation of Hs-MANF is reduced by sulfatide dose-dependently. Only N-terminal peptides were detected for MANF. **d** Western blot showing that the uptake of Hs-MANF by *C. elegans* is dependent on sulfatide but independent of developmental stages and the feeding bacterium OP50.

**Supplementary Figure** **4** **Hs-MANF attenuates caspase-mediated apoptosis in HEK293T and H9C2 cardiomyocyte cells by sulfatide binding.** **a** Effects of hypoxia/reperfusion on the Caspase-3 processing by Western blot in HEK293T cells. **b** Effects of hypoxia/reperfusion on the caspase-3 processing by Western blot in H9C2 cardiomyocyte cells. **c** Quantification of cell death rates of H9C2 cells after hypoxia /reperfusion with treatment of Hs-MANF or Hs-MANF(K112L) mutants. N=4 biological replicates, *** P<0.001, ANOVA. Error bars: S.D.

**Supplementary Figure** **5** **MANF and sulfatide interaction promotes cellular uptake of MANF not sulfatide degradation. a** Western blot using lysates from HEK293T cells treated with Hs-MANF or Hs-MANF (K112L) conditioned PBS in the absence or presence of sulfatide or over-expression of the gene CST, encoding a sulfatide biosynthetic enzyme (HSP90 used as loading control). **b** Quantification of sulfatide levels after extraction from Hs-MANF-overexpressing or KO HEK293T cells. N=3 biological replicates, *** P>0.05, non-significant. Error bars: S.D. **c** Western blot using lysates from HEK293T cells and MANF KO cells (clone C6) with indicated treatment of tag-free MANF and V5-tagged MANF from conditioned media of overexpressing lines.

**Supplementary Figure** **6 Effects of hypoxia and pH on MANF secretion and interaction with sulfatide.** **a** Western blot of Hs-MANF::V5 secreted from HEK293T stable cell lines with treatment of hypoxia, serum starvation and Tg. **b** WB of Hs-MANF::V5 from cells cultured under OGD (oxygen, glucose deprivation) were treated with Brefeldin A (BFA, 50 ng/ml) or DMSO (vehicle). **c** Size exclusion chromatography measurements showing optimal pH at about 6.5 for sulfatide and MANF interaction.

**Supplementary Figure** **7 Sulfatide binding is required for specific endocytosis of MANF. a** Western blot of uptaken Hs-MANF with treatment of the sulfatide antibody O4 or sulfatase. Re: the medium containing O4 antibody (0.01 mg/mL) or sulfatase (20 µg/mL) removed before adding the conditioned medium. Ke: O4 antibody or sulfatase kept during medium treatment. **b** Western blot of MANF::V5 after being purified from the medium and added to target cell culture. “-Sf” indicates sulfatide sequestration by O4. “+Sf” indicates addition of exogenous sulfatide to the medium. IgG was used as control. **c** Western blot of Hs-MANF at different times upon sulfatide addition to the target cell medium. **d** Western blot of MANF::V5, ADIPOQ::V5 or C1ORF123::V5 using media from overexpressing stable lines with or without sulfatide for 24 hrs. **e** Western blot of Hs-MANF::V5 with 1 h pre-incubation of endocytosis pathway inhibitors (Cytochalasin, 0.4 µM; Filipin, 0.5 µg/ml; Chlorpromazine, 5 µg/ml and dynasore, 40 µM).


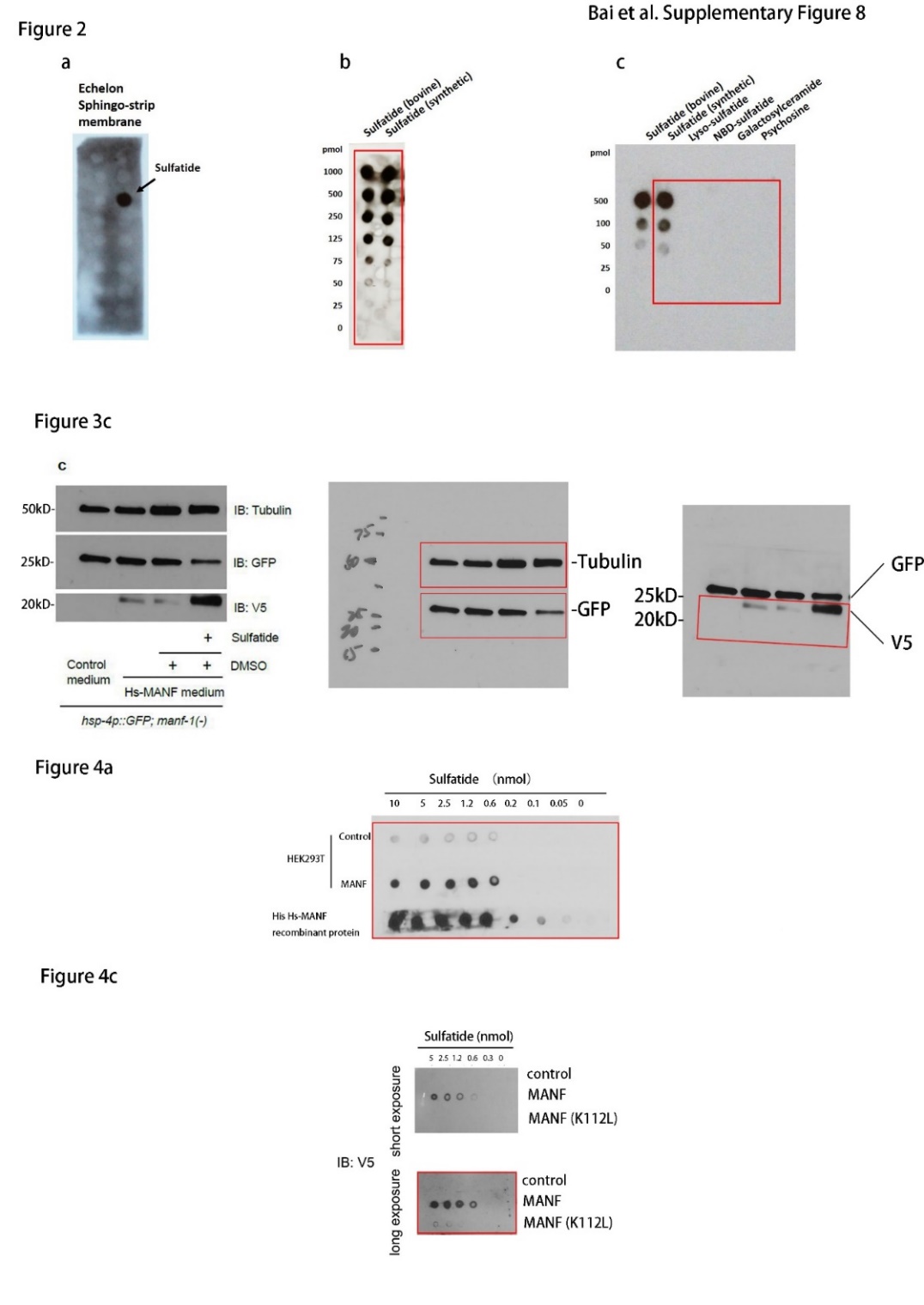


**Supplementary Figure 8 Uncropped scans of blots from indicated figures.**


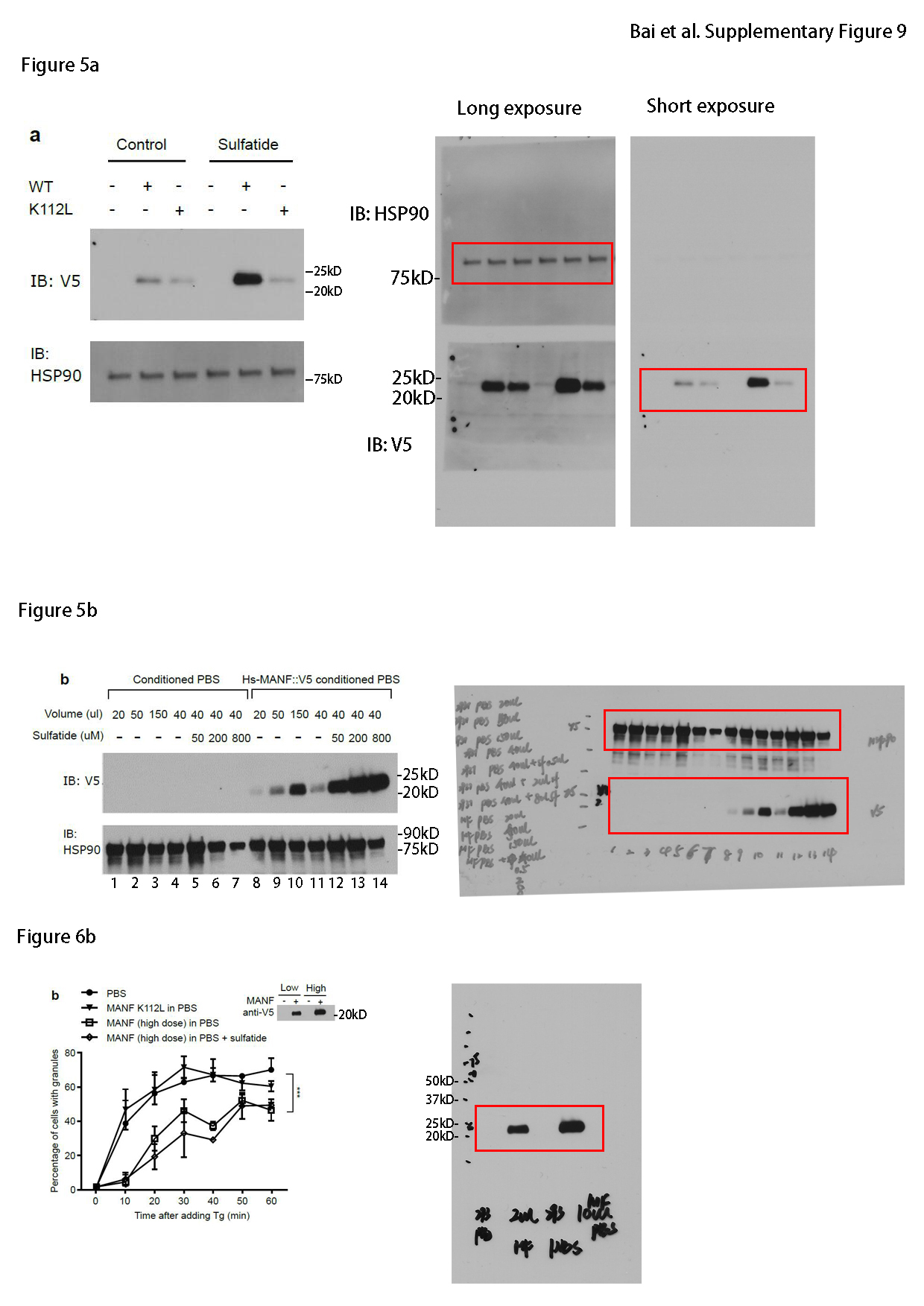


**Supplementary Figure 9 Uncropped scans of blots from indicated figures.**
